# Abnormal Exercise Gas Exchange Before Pulmonary Emboli Diagnosis

**DOI:** 10.1016/j.mayocpiqo.2024.10.001

**Published:** 2024-11-13

**Authors:** Timothy Edwards, Elisabet Børsheim, Andrew R. Tomlinson

**Affiliations:** a Arkansas Children’s Nutrition Center, Little Rock, AR; b Arkansas Children’s Research Institute, Little Rock, AR; c Department of Pediatrics and Department of Geriatrics, University of Arkansas for Medical Sciences, Little Rock, AR; d Institute for Exercise and Environmental Medicine, Texas Health Presbyterian Hospital Dallas and University of Texas Southwestern Medical Center, Dallas, TX

## Abstract

A 20-year-old male underwent diagnostic testing due to unexplained shortness of breath and chest discomfort. He had no previous medical problems and was not taking any medications. Initial evaluations included cardiopulmonary exercise testing (CPET), which yielded results that were reported as normal. However, over the following 2 months, his symptoms worsened considerably, including dyspnea with climbing stairs and then hemoptysis. Seeking urgent medical care, he presented to the emergency department, where he underwent further testing and was admitted to the hospital. Computed tomography angiogram reported bilateral pulmonary emboli. His parents requested a second opinion regarding the analysis of the CPET data, which revealed previously overlooked abnormalities. This overlooked data delayed pulmonary embolism diagnosis, and the patient ultimately required bilateral pulmonary thromboendarterectomy. In this case, we describe the hallmark signs of pulmonary vascular disease seen during CPET and offer clinical pearls to aid in timely detection.

Pulmonary thromboembolic disease, including pulmonary embolism (PE), affects an estimated 500,000-600,000 people annually in the United States, with ∼200,000-300,000 deaths per year.^1^ Although PE is more common in older individuals, it can also occur in young, healthy adults, often presenting diagnostic challenges due to nonspecific symptoms. The incidence in younger populations is lower, but early detection is critical because of the risk of life-threatening complications such as long-term thromboembolic pulmonary hypertension (CTEPH). The CTEPH incidence in young adults per se is less well described; however, CTEPH after short-term PE in those without major comorbidities has been found to be in the range of 1%-3%.^2^

The cardiopulmonary exercise testing (CPET) enables investigators to assess the responses of the cardiovascular and ventilatory systems to incremental metabolic stress. Through the measurement of oxygen uptake ($\dot{V}$O_2_), carbon dioxide elimination ($\dot{V}$CO_2_), and minute ventilation ($\dot{V}$E), a comprehensive evaluation of the physiologic response to exercise is achievable. Therefore, CPET provides investigators with the means to differentiate between normal and abnormal response patterns characteristic of various disease states.

The CPET has been shown to be a valuable noninvasive diagnostic tool capable of accurately differentiating between normal responses and either CTEPH or long-term thromboembolic disease, with a sensitivity and specificity of 92% and 83%, respectively, in predicting a diagnosis of CTEPH.^3^ Important differences are seen in dead-space ventilation to tidal volume ratio, minute ventilation to carbon dioxide ratio ($\dot{V}$E/$\dot{V}$CO_2_), and end-tidal carbon dioxide pressure (PETCO_2_). These variables provide key information on the ability of the lungs to effectively facilitate gas exchange between the circulatory system and the environment.

In those with short-term or long-term unexplained shortness of breath, CPET should be considered as a first-line tool aiding in the evaluation of pulmonary gas exchange efficiency during exercise. This case describes overlooked abnormal exercise gas exchange responses suggestive of pulmonary vascular disease (PVD). Despite normal trans-thoracic echocardiogram, cardiorespiratory fitness level, and resting pulmonary function, our second opinion CPET analysis found abnormal exercise gas exchange responses were present 2 months before PE diagnosis. The importance of this finding is seen in the eventual diagnosis of bilateral PE, followed by CTEPH, and ending with bilateral pulmonary thromboendarterectomy (PTE). Thus, this case reports the valuable role of CPET in revealing PVD and the importance of early recognition of abnormal exercise gas exchange, as timely intervention is needed to prevent life-threatening outcomes.

## Case Report

### Physical Examination Findings

In March 2023, a 20-year-old male presented to an urgent care facility complaining of several months of shortness of breath and intermittent chest discomfort. He was given antibiotics for an assumed upper respiratory tract infection. The following month, April 2023, diagnostic testing was scheduled with an outside testing facility because of no improvement in symptoms. He presented to the testing facility with a heart rate of 81 beats/min, blood pressure of 120/74 mm Hg, respiratory rate of 14 breaths/min, and oxygen saturation of 97% on room air. He had no known previous or current medical history and was not taking any prescription medications other than the antibiotics from the previous month. To investigate the origins of his symptoms, a trans-thoracic echocardiogram and CPET were performed.

### Echocardiogram and CPET Assessment

A trans-thoracic echocardiogram reported normal left ventricular function with an ejection fraction of 55%-60%, and normal left and right ventricle anatomical measures and valve function. All pulmonary function tests were within reference ranges (spirometry, diffusion capacity of the lung for carbon monoxide, nitrogen washout, and maximum voluntary ventilation). Resting electrocardiogram (ECG) found sinus rhythm with an incomplete right bundle branch block. An incremental CPET on a cycle ergometer was performed. He stopped due to leg fatigue after reaching a respiratory exchange ratio ($\dot{V}$CO_2_/$\dot{V}$O_2_) of 1.07, peak heart rate of 174 beats/min (87% of predicted), and a $\dot{V}$O_2peak_ of 37.6 mL·kg^−1^·min^−1^ (cardiorespiratory fitness; 87% of predicted). Oxygen saturation at peak exercise was 96%. Exercise ECG was negative for both dysrhythmia and myocardial ischemia. There were no abnormal findings reported.

### Computed Tomography Angiogram

**Figure 1:**
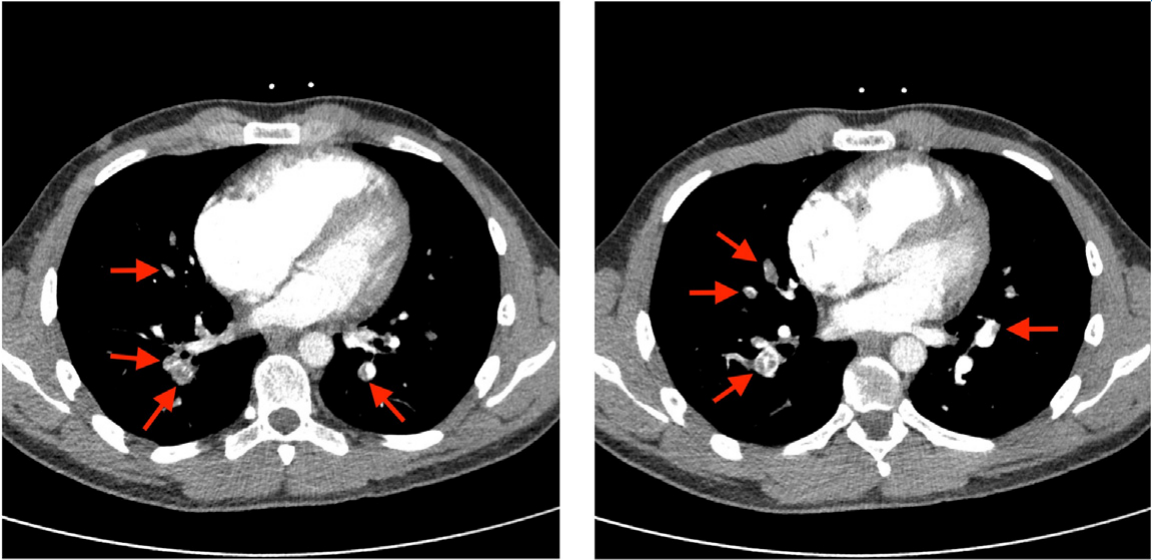
Axial contrast-enhanced Computed Tomography scan reporting bilateral pulmonary emboli.

In June 2023, 2 months after the reported normal CPET, his dyspnea had worsened with climbing stairs and then hemoptysis. He was admitted to the emergency department where a computed tomography (CT) angiogram was ordered. Findings from the CT angiogram found that bilateral PE was present (Figure 1), and he was initiated on apixaban.

### Second Opinion CPET Analysis

**Figure 2:**
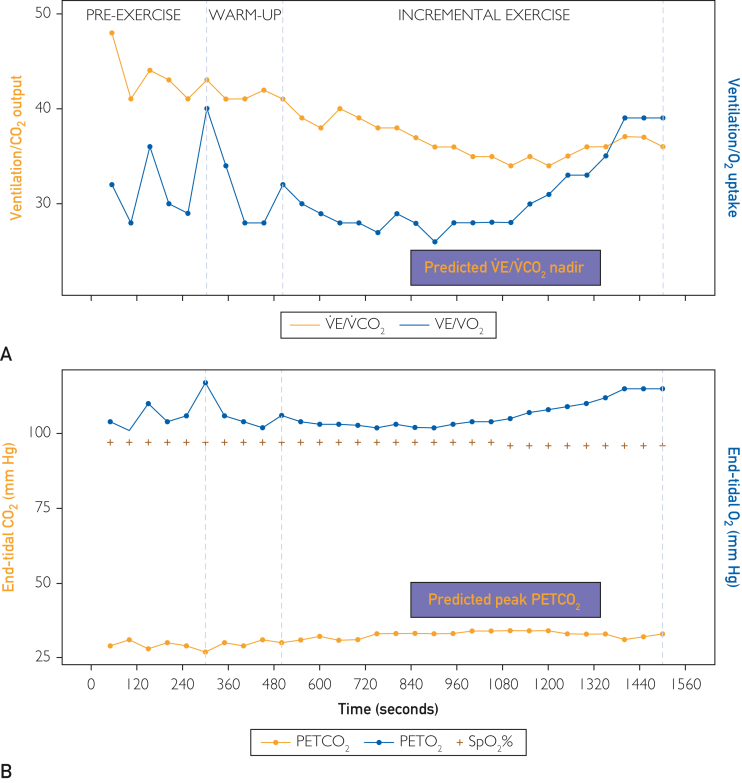
Key cardiopulmonary exercise testing data. Plot A, note the elevated lowest ratio of ventilation to CO_2_ elimination ($\dot{V}$E/$\dot{V}$CO_2_ nadir) of 34 compared with his predicted range of 23.9±2.1. This abnormal response reflects the compensatory $\dot{V}$E response to an increased dead-space fraction. Plot B, note end-tidal CO_2_ pressure (PETCO_2_) is reduced at rest and at its peak. Normal resting PETCO_2_ values at sea level are 36-42 mm Hg, and normally increase as much as 3-8 mm Hg by mid-exercise, whereas his was 29 mm Hg at rest with a peak of 34 mm Hg. Although his PETCO_2_ increases with incremental exercise, the peak value is reduced and reflects increased dead-space ventilation. Shaded areas = age and sex matched predicted ranges.^4^^,^^5^

The following month, July 2023, after the PE diagnosis, the parents requested a second opinion analysis of the CPET. Our analysis revealed overlooked abnormal gas exchange responses (Figure 2).

### Pulmonary Hypertension Clinic

In October 2023, a new trans-thoracic echocardiogram was performed, which indicated pulmonary hypertension was present along with an enlarged right ventricle. The following month, November 2023, a lung perfusion scan revealed multiple segmental mismatched perfusion deficits. He was diagnosed with CTEPH, and plans were made for bilateral PTE.

### Bilateral PTE

In December 2023, he successfully underwent bilateral PTE. Because of the possibility of anatomical thoracic outlet syndrome as the cause of the PE, rib resection was performed 4 months later in April 2024.

## Discussion

**Figure 3:**
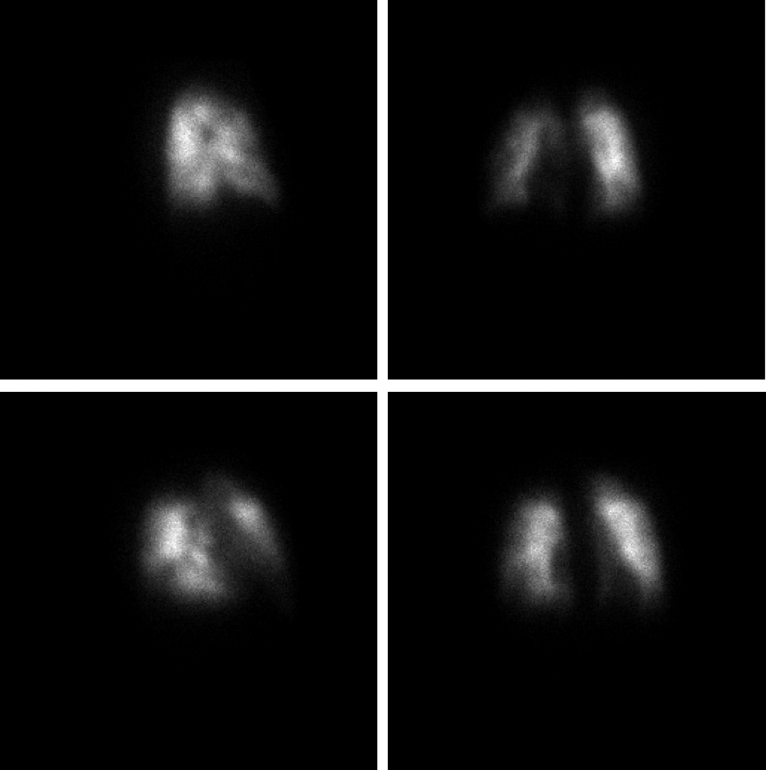
Planar perfusion scintigraphy showing multiple segmental mismatched perfusion deficits.

Our second opinion analysis of exercise gas exchange suggested a pattern of ventilatory response concerning for either hyperventilation or increased dead-space ventilation (ventilation-perfusion mismatch). This pattern can be seen with PVD, including pulmonary hypertension. These abnormalities were identified through an elevated ratio of $\dot{V}$E/$\dot{V}$CO_2_ and a reduced PETCO_2_. A normal value for $\dot{V}$E/$\dot{V}$CO_2_ nadir in males aged 20-30 years is 23.9±2.1^4^ (his was 34), with normal resting PETCO_2_ values at sea level ranging from 36-42 mm Hg and normally increasing as much as 3-8 mm Hg by mid-exercise^5^ (his was 29 mm Hg at rest with a peak of 34 mm Hg) (Figure 2). These findings suggest possible PVD, including from pulmonary hypertension or from pulmonary emboli, causing an abnormal distribution of pulmonary blood flow. Elevated $\dot{V}$E/$\dot{V}$CO_2_ responses, as described by Sun et al,^4^ often indicate high dead-space ventilation, typically due to poor perfusion, and this was later confirmed by perfusion deficits seen on his lung perfusion scan (Figure 3). Of importance, hyperventilation and increased dead-space ventilation produce similar patterns of $\dot{V}$E/$\dot{V}$CO_2_ and PETCO_2_ and can only be definitively differentiated by measurement of arterial blood gases. Unfortunately, no arterial blood gas measures were collected by the testing facility during the initial CPET.

Interestingly, our case exhibited an uncommon presentation of the effect of PE on cardiorespiratory fitness. Typically, similar cases have reported reduced cardiorespiratory fitness ($\dot{V}$O_2peak_) during CPET.^6^, ^7^, ^8^, ^9^ Surprisingly, our case had a normal cardiorespiratory fitness level (87% predicted) along with normal peak O_2_ pulse (sometimes used as a surrogate for stroke volume), anaerobic threshold, Δ$\dot{V}$O_2_/Δworkload, and peak heart rate, suggesting normal cardiovascular function. However, significant abnormalities in gas exchange responses were seen, specifically an elevated $\dot{V}$E/$\dot{V}$CO_2_ nadir and reduced PETCO_2_. These abnormal responses are consistent with previous reports involving PE.^6^, ^7^, ^8^, ^9^ Contrary to other reports, the normal $\dot{V}$O_2peak_ seen in this case was likely due to either a relatively mild disease state at the time of testing (2-months before diagnosis) or higher baseline fitness before disease onset.

It is likely that if the initial CPET had been more thoroughly analyzed, the PE may have been found earlier, which would have allowed treatment to be started sooner. However, this overlooked data contributed to a delayed diagnosis and risked a potentially fatal cardiopulmonary event. Notably, although the echocardiogram and CPET cardiovascular parameters were normal, the abnormal pulmonary gas exchange responses pointed to underlying issues in the pulmonary circulation. Therefore, this case underscores the importance of fully reviewing all data obtained from CPET, as abnormal gas exchange responses may serve as an early indicator of PVD, including PE, even in cases where traditional measures of cardiorespiratory fitness remain within normal ranges. This nuanced perspective emphasizes the necessity of a comprehensive CPET interpretation, extending beyond conventional fitness metrics for an accurate diagnosis. For preventive measures, these abnormal responses and the unfortunate patient outcome were communicated to the testing facility that performed the CPET to prompt the necessary re-education to improve the patient care process.

PE typically occurs when deep vein thrombus dislodges and enters the pulmonary circulation, thus affecting ventilation and perfusion matching.^10^ A recent review of short-term PE outlines criteria for classifying very low-risk patients (age <50 years, heart rate <100 beats/min, oxygen saturation >94%, no recent operation or trauma, no previous venous thromboembolism event, no hemoptysis, no unilateral leg swelling, and no estrogen use).^11^ Diagnosis may more likely be delayed in those otherwise at very low risk or with more long-term symptoms.

Early diagnosis of CTEPH is often challenging, with one report reporting a mean diagnosis delay of 14 months.^12^ One of the major barriers to early CTEPH diagnosis is the nonspecific symptoms it produces. CPET, with its capability to assess the efficiency of pulmonary gas exchange, serves as a useful tool in CTEPH detection. This usefulness was seen in the present case, where a trans-thoracic echocardiogram, pulmonary function testing (including diffusion capacity of the lung for carbon monoxide), and ECG did not detect any abnormalities. It would not have been known until CPET gave the clue of a ventilation-perfusion mismatch that the cause of his symptoms stemmed from the pulmonary vascular system. In cases involving PE,^6^, ^7^, ^8^, ^9^ common abnormal CPET parameters included elevated $\dot{V}$E/$\dot{V}$CO_2_ and reduced PETCO_2_. The $\dot{V}$E/$\dot{V}$CO_2_ relates the minute ventilation to the rate of carbon dioxide elimination and is often viewed as a marker of ventilatory efficiency and can be affected by PVD and ventilation-perfusion mismatch. The PETCO_2_ is generally similar to arterial partial pressure of carbon dioxide but can be reduced by increased ventilation-perfusion mismatch. This shared abnormality highlights the sensitivity of these parameters to the pathophysiology of PE affecting ventilation-perfusion matching. Therefore, in individuals at very low risk or with long-term symptoms, CPET may have advantages as a first-line test for value-based care. However, the effectiveness of CPET as a diagnostic tool relies on experienced interpretation, emphasizing the importance of thorough analysis for accurate diagnosis, and timely treatment initiation.

### Clinical Course

The patient underwent initial assessments of echocardiogram and CPET in an attempt to identify the cause of his unexplained shortness of breath and chest discomfort. Despite both tests reporting normal results, his symptoms worsened over the next 2 months, reaching a point where he struggled to climb a flight of stairs and noticed blood in his sputum. Seeking urgent medical attention, he presented to the emergency department, where CT angiography with contrast revealed bilateral PE, and he was initiated on apixaban.

During the treatment and management period, further evaluation confirmed the presence of long-term thromboembolic pulmonary hypertension. Because of the limited effectiveness of anticoagulation therapy, the decision was made to proceed with PTE operation. This operation was successful, and 4 months after PTE he also underwent a rib resection for thoracic outlet syndrome (interestingly, his mother had an upper extremity embolism at age 22 and underwent a rib resection for thoracic outlet syndrome at that time). At the 3-month follow-up appointment, he reported substantial improvement in his symptoms and quality of life.

### Clinical Pearls

(1) Pulmonary embolism may be underdiagnosed as chest pain and shortness of breath are common symptoms in various serious cardiopulmonary conditions, and benign causes. These symptoms in young, healthy individuals may lead to numerous diagnostic tests that often fail to reveal clear pathology. However, CPET with evaluation of gas exchange is a noninvasive test capable of detecting abnormal pulmonary vascular responses in the early stages of the disease, even before the progression to pulmonary hypertension.
(2) The characteristic pattern of PVD during CPET manifests as reduced PETCO_2_ and elevated $\dot{V}$E/$\dot{V}$CO_2_ response. Similar patterns can be seen with hyperventilation and sometimes in patients with heart failure. The CPET can help in ruling out specific organ system involvement, as found in the present case. It is imperative to consider all abnormal CPET responses, even in cases where the patient maintains normal cardiorespiratory fitness, cardiac function, and resting pulmonary function (including lung diffusing capacity) at the time of testing.
(3) The CPET may serve as a valuable first-line investigative tool in individuals with dyspnea, but at very low risk of PE, as it enables early detection of PVD, facilitating the possibility of prompt initiation of appropriate medical therapy. Thorough training in CPET interpretation is essential for clinicians to ensure the delivery of value-based health care.

## Potential Competing Interests

The authors have no conflicts of interest to disclose.
